# What factors do parents/caregivers think impact change in family therapy for anorexia nervosa?: a qualitative study

**DOI:** 10.1186/s40337-025-01368-x

**Published:** 2025-11-06

**Authors:** Paige James, Julian Baudinet, Ivan Eisler, Anna Konstantellou, Mima Simic, Anna Oldershaw, Ulrike Schmidt

**Affiliations:** 1 Salomons Institute for Applied Psychology, Canterbury Christ Church University, Tunbridge Wells, UK; 2 Centre for Research in Eating and Weight Disorders (CREW), Institute of Psychiatry, Psychology and Neuroscience, King’s College London, De Crespigny Park, London, SE5 8AZ UK; 3 Maudsley Centre for Child and Adolescent Eating Disorders, South London and Maudsley NHS Foundation Trust, De Crespigny Park, Denmark Hill, London, SE5 8AZ UK; 4 Kent and Medway All Age Eating Disorders Service, North East London NHS Foundation Trust (NELFT), Maidstone, UK; 5 Adult Eating Disorders Service, South London and Maudsley NHS Foundation Trust, De Crespigny Park, Denmark Hill, London, SE5 8AZ UK

**Keywords:** Anorexia nervosa, Family therapy, Family-based treatment, Maudsley family therapy, Reflexive thematic analysis

## Abstract

**Background:**

While the efficacy of family therapy for adolescents with anorexia nervosa is well documented, the process of change across treatment is less well understood. Emerging research has looked at the young person experience, however, little is known about the parent/caregiver perspective. This study aimed to understand factors that parents/caregivers perceive as facilitating change in family therapy for anorexia nervosa (FT-AN).

**Methods:**

Twenty-three parents/caregivers of young people (age 12–18 years) with anorexia nervosa who had completed FT-AN participated in individual semi-structured interviews online. Interviews were transcribed verbatim and analysed using reflexive thematic analysis.

**Results:**

Five interconnected themes were generated: *Not alone, Strong foundations, Commitment, Both/and rather than either/or,* and *Strengthening family connection*. Parents /caregivers highlighted the importance of collaboration—both within the family and with the clinical team—in building a support network. This collaborative foundation was seen as central to facilitating change, initially through structure and boundaries, and later through increased flexibility and safe risk-taking. Maintaining a life outside the illness and ensuring a balance between physical and emotional needs across all stages of treatment emerged as critical to the recovery journey.

**Conclusion:**

This qualitative study explored parental experiences of supporting a young person through FT-AN. Themes generated in this study closely mirror the change processes reported by young people and align with the theoretical underpinnings of FT-AN. Parents reported that change was supported through collaboration with knowledgeable clinicians, setting clear expectations and reduced isolation. A holistic, person-centred approach to treatment that considered life outside the illness was considered as key in promoting change and building commitment from the young person and family. Additionally, finding the right balance in safe risks taking, flexibility within the approach and gradual spacing out of sessions were all described as key to promoting change.

**Supplementary Information:**

The online version contains supplementary material available at 10.1186/s40337-025-01368-x.

## Abstract (plain language summary)

Family therapy for anorexia nervosa (FT-AN) is a first-line recommended treatment for adolescents with anorexia nervosa. While it is helpful for most, there are many who require more or something different to reach recovery. This study aimed to understand factors that parents/caregivers perceive as impacting change in FT-AN. Twenty-three parents/caregivers of young people (age 12-18 years) with anorexia nervosa who had completed FT-AN participated in individual semi-structured interviews. Analysis of transcripts using reflexive thematic analysis generated five interconnected themes: 1) Not alone, 2) Strong foundations, 3) Commitment, 4) Both/and rather than either/or, and 5) Strengthening family connection. Parents/caregivers highlighted the importance of collaboration—both within the family and with the clinical team—in building a support network. This collaborative foundation was seen as central to facilitating change, initially through structure and boundaries, and later through increased flexibility and safe risktaking. Parents/caregivers reported that change was supported through collaboration with knowledgeable clinicians, setting clear expectations and reduced isolation. A holistic, person-centred approach to treatment that considered life outside the illness was considered as key in promoting change and building commitment from the young person and family.

**Supplementary Information:**

The online version contains supplementary material available at 10.1186/s40337-025-01368-x.

## Introduction

Despite evidence and international guidelines supporting anorexia nervosa focused family interventions as the first line recommended treatment for adolescent anorexia nervosa [1], it is not effective for a significant minority of young people [2]. Furthermore, relatively little is understood about how these treatments work [3–5]. The most consistent finding is that early weight gain is predictive of end-of-treatment outcomes in manualised Family-Based Treatment [6–8]; however, beyond this, the data are mixed. Some studies also suggest parental self-efficacy around mealtimes may be a treatment mechanism [9, 10]. Understanding treatment mechanisms will aid the formulation process [4, 11] and future treatment developments, enabling adaptation to the needs of each individual and their family in an evidence informed way.

Several models of family therapy for anorexia nervosa exist, including Maudsley family therapy for anorexia nervosa (FT-AN; [12]), Family Based Treatment (FBT; [13]) and multi-family therapy (MFT; [14]). While differences between these models exist, they have recently been described as more similar than different (cf [15] for a discussion of the models). Multi-family therapy for anorexia nervosa (MFT; [14]) is a group-based adaptation of FT-AN. It is based on the principles of FT-AN and can be delivered alongside individual FT-AN sessions or as a standalone treatment (cf [16–19] for reviews).

Increasingly, data are emerging regarding treatment experience of FBT and FT-AN [20, 21], perceived change processes [22], as well as moderators and mediators of treatment outcome [23–25]. Qualitative data on the young person and parent experience of family therapies for anorexia nervosa suggest that treatment can sometimes be experienced as inflexible and too behaviourally focused [21]. Conti and colleagues [20] interviewed families in which the young person experienced continued distress post treatment or who dropped out of FBT. For this group, they found that the young person reported a sense of losing their voice and identity during treatment. This was particularly noted early in therapy where parents are encouraged to take a more directive role in supporting re-feeding. An early over-focus on parents has been perceived as contributing to treatment non-completion by a lack of attention to building distress tolerance skills for the young person [26].

A recent review and meta-synthesis of 15 qualitative studies of FBT and FT-AN [22] generated six main themes describing facilitators of and barriers to change across family therapy: (1) A holistic focus on the young person’s overall development, (2) The therapeutic relationship as a vehicle for change; (3) The therapist’s narrow focus confined to a “script” on food intake and weight gain and its impact on emotional attunement, (4) A disempowering therapeutic context, (5) Externalisation of the eating disorder (ED) and (6) The importance of family involvement. The authors noted that positive changes are perceived to occur when a holistic approach is taken for the young person’s overall development including psychological, emotional, social and physical wellbeing within the context of a containing, positive therapeutic relationships. This fits with the concepts of epistemic trust (the extent to which someone trusts and can consider information from others [27]) and ‘relational containment’, a term coined by Wallis et al. [28], to describe how young people and families can move from a place of distress to one of hope and effective recovery-oriented change within the context of a supportive network.

Of note, broader research in the field of eating disorders and beyond suggests that the therapeutic alliance is an important predictor of treatment outcome [29–31]. However, data from within the FBT/FT-AN literature are more mixed. Some FBT studies have found that therapeutic alliance was not a predictor of outcome [32, 33], whereas an FT-AN study did [34]. In their review, Cripps and colleagues [22] reported that positive change was hindered by inflexibility in the treatment approach, a narrow focus on food-intake and weight, as well as the neglect of family difficulties, emotional experiences, and psychological factors.

A recent study (N = 15) explicitly explored how change is perceived to occur in FT-AN from the young person’s perspective [35]. Results suggest that trusting relationships with family members and professionals are a key factor in promoting change and being able to tolerate distress associated with weight gain and recovery. In the context of these trusting relationships, young people spoke about the importance of personally acknowledging (often privately) that they are unwell and need to make a change, while staying connected to life outside the illness and facing, rather than trying to avoid, difficulties. They also spoke about the importance of shifting treatment focus away from food and eating and reducing session frequency once behaviour change begins. They described this as helping to keep momentum and motivation for recovery and to prepare for discharge [36].

Some of these themes align more generally with the broader literature on recovery from anorexia nervosa. For example, qualitative data on the adult experience describe the importance of ‘seeing through the facade’ [38], 'seeing the dangers' and 'inching out' anorexia [39] as part of the recovery process. Similarly, identity formation and/or reclamation is also described as a key aspect of this process [40].

While there is preliminary understanding of the parent and clinician experiences of MFT [16, 23, 37–43], data have not yet been reported on the parent experience of FT-AN. This study aims to address this by exploring parent and caregiver experiences of what factors they perceive to be influencing change during FT-AN.

## Method

Ethics approval was granted for this project by the Stanmore Research Ethics Committee London (IRAS: 234354; REC: 20/LO/0839). All participants provided written informed consent. This included consent to use de-identified quotes from transcripts for publication.

### Sample

Parents were eligible to participate in the study if (a) their young person had a diagnosis of anorexia nervosa or atypical anorexia nervosa and (b) they had received FT-AN as part of their outpatient treatment at the Maudsley Centre for Child and Adolescent Eating Disorders (MCCAED). Potential participants were identified at the point of discharge from treatment by the clinical team, who then made initial contact about the study. Once consent to contact from the research group was received, up to three attempts at contact were made per person.

While there were no specific exclusion criteria, participants needed an adequate level of English to understand the participant information sheet, consent form and to participate in the interview. All participants answered questions regarding perceived factors, both positive and negative, influencing recovery-oriented change in therapy and potential elements that had been missing or unhelpful in therapy.

### Recruitment and design

All participants who completed FT-AN at MCCAED (cf [44, 45] for service description and outcomes) between July 2022 and February 2023 were approached to participate in the study.

All interviews were conducted via video-call by authors JB (male, white Australian, clinical psychologist, PhD, extensive experience of FT-AN) and AK (female, assistant psychologist, PhD, extensive theoretical knowledge, but no clinical experience, of FT-AN). JB had been involved in treatment delivery for some participants, therefore interviews for these families were completed by AK. Each interview lasted approximately 60 min (range = 12–80 min) with only the interviewer and the participant present. Interviews were recorded and then transcribed verbatim. All interviews followed the same topic guide. Transcriptions were not returned to participants for review.

Participants were informed at the beginning of their interview that the aim of the research was to explore their experience of how they perceived change to occur in treatment.

### Data analysis

All interview transcripts were analysed using reflexive thematic analysis [46]. This process emphasises the use of deep reflection, researcher subjectivity and a recursive coding process, whereby two or more rounds of coding may be completed, prior to generating themes. An inductive approach to analysis was utilised in an attempt to generate themes that fitted closely to the data. The six phases of reflexive thematic analysis were followed (see supplementary material for details and for reflexivity statements of analysing authors). Analysis was completed by authors PJ and AK, and supervised by JB. After initial familiarisation with the data, both analysers then independently generated codes. Preliminary themes were generated iteratively across two separate one-hour meetings before reaching consensus on final themes.

A critical realist epistemological stance was adopted, where experience and meaning are considered subjective interpretations of reality that are influenced by social and cultural contexts [47]. Given that different meanings are generated by different researchers during the reflexive analysis process, the concept of data saturation was not used in line with current recommendations [48]. Analysers drew on existing knowledge of FT-AN, systemic theory and systemic literature. Some concepts were expected to emerge (e.g. the importance of trusting relationships and the need for firm consistency during the early stages of FT-AN). Coding was not constrained by these expectations, although it is recognised that this knowledge will have, without a doubt, influenced the findings. Throughout the analysis process, PJ maintained a reflexive research diary to reflect on researcher bias and preconceived expectations.

#### Diagnostic assessment and screening

Eating disorder diagnosis was established at initial assessment through clinical interview and self-report data from the Development and Well-being Assessment (DAWBA; [49]). The DAWBA is a structured diagnostic tool that generates predicted diagnoses for children and adolescents based on criteria from the DSM-5 (American Psychiatric Association, [50]) and ICD-10 (World Health Organization, [51]). The DAWBA has good reported validity [52].

### Treatment description

Participants received outpatient FT-AN [12, 53]. FT-AN follows four phases, (1) engagement and development of therapeutic alliance, (2) helping the family manage the eating disorder symptoms, (3) exploring issues of individual and family development, and 4) endings and discussion of future plans. Sessions begin weekly, moving to fortnightly and even less frequently in the later stages of treatment. Changes in frequency of sessions are agreed with the family based on treatment response.

The emphasis in the initial phase is on engaging the whole family including the young person and purposefully leveraging the therapist and team expertise to create a safe, containing context for the forthcoming treatment. This is followed in the next phase with a focus on practical support to manage symptoms, gain weight as needed and build distress tolerance. The therapist continues to seek opportunities to strengthen the engagement with the young person, explore their motivation to change and validate their concerns.

Later phases are more individualised. Typical themes within the later stages of FT-AN include handing back responsibility (parents to young person; therapist to family), navigating school and peer relations, regaining independence with eating and tolerating uncertainty. The final phase is an opportunity to review the family journey through treatment and explore current/future family life without an eating disorder (cf [18] for a more detalied recent description the FT-AN model).

## Results

Parents/caregivers from a total of 46 families were approached to participate. Of these, at least one parent from 19/46 families (41.3%) consented and participated, 10/46 (21.7%) actively declined, 12/46 (26.1%) passively declined (provided initial consent or requested further information but were unreachable or unavailable to complete an interview thereafter), and 5/46 (10.9%) could not be reached with a maximum of three attempts at contact. Reasons for non-participation included being too busy, not being interested in the study and not wanting to revisit the illness/treatment journey. Families were approached regardless of whether they were deemed to have had a good outcome or not.

### Demographic data

Twenty-three parents/caregivers (18 mothers, 5 fathers) from 19 families comprised the final sample in this study. All parents/caregivers identified as cisgendered. The majority identified as White British (n = 16, 69.6%) with the remaining participants identifying as White Other (n = 4, 17.4%), and non-White British (n = 3, 13.0%). No participant identified as neurodivergent or living with a disability. Mean age at interview was 49.91 years (sd = 5.89). Five (5/23, 21.7%) parents were from separated families.

Most parents/caregivers described FT-AN as helpful (21/23, 91.3%) although some did not (2/23, 8.7%). The two participants from different families who did not report FT-AN to be helpful said their young person had experienced weight restoration, but little or no psychological recovery. Both felt there was not enough focus in sessions for psychological change to occur but spoke of the elements they thought would have been helpful for change.

The mean age of young people from the 19 families was 15.37 years (sd = 1.50). Most young people (13/19, 68.4%) met DSM-5 criteria for anorexia nervosa (restrictive subtype = 11, binge-purge subtype = 2), and six (6/19, 31.6%) for Other Specified Feeding or Eating Disorder (OSFED, atypical anorexia nervosa). Mean percentage median Body Mass Index (%mBMI) was 84.5% (sd = 9.04) at assessment and 94.2% (sd = 7.43) at discharge. There was a significant increase from baseline to discharge [*t*(17) = 5.24, *p* < 0.001]. The participating parents were of young people who had a relatively heterogeneous treatment course. The mean duration of treatment was 7.4 months (sd = 3.53); however, it ranged from 3 to 18 months. The ending of treatment was based on a consensus agreement between the family and therapist, reached when the family no longer felt they needed further support and the clinical team deemed it safe to discharge. The mean duration from the final FT-AN session to interview was 4.49 months (sd = 5.72, range = 0.07–17.36).

Young people from participating (n = 19) and eligible non-participating families (n = 27) did not significantly differ in age [n = 45, mean difference = 0.14 years, *t*(44) = 0.07, *p* = 0.78], treatment duration [n = 45, mean difference = 1.61 months, *t*(44) = 1.45, *p* = 0.16], weight at initial assessment [n = 45, mean difference = 1.89%mBMI, *t*(44) = 0.71, *p* = 0.48] or discharge [n = 43, mean difference = 1.2%mBMI, *t*(42) = 0.56, *p* = 0.58], tested using independent samples t-tests.

### Qualitative findings

**Fig. 1:**
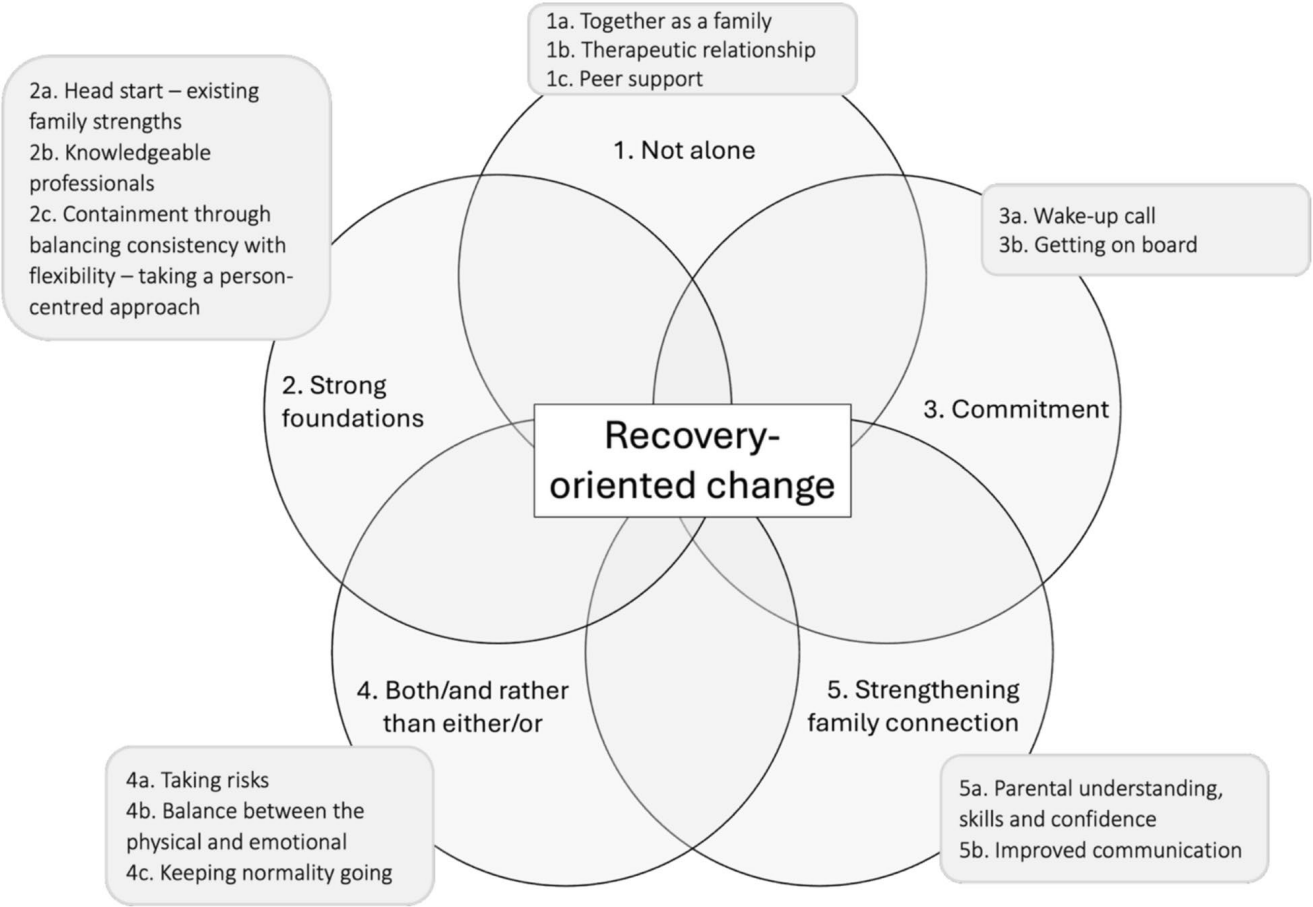
Interconnected themes and sub-themes generated

Factors perceived to influence change, both within and outside of therapy, were grouped into five main themes and thirteen subthemes. Themes and subthemes are described below and are presented in Fig. 1.

1. Not alone

The first main theme *‘not alone’* captured the idea that feeling connected to others in the journey is key for fostering change. This was described as important for young people and parents. This encompasses subthemes of the experience of coming *‘together as a family'*, receiving the right support from a professional via the *‘therapeutic relationship’* and engaging with *‘peer support’*.

*1a. Together as a family.* Almost all participants spoke of the importance of going through therapy together. Many felt that if the young person had been alone in treatment, progress would have been slower and may not have occurred at all. This was described by most participants, including the two who described FT-AN as unhelpful. There was a strong sense that being together created a united front in managing illness behaviours and symptoms.

*“I think the whole sitting down and having meals and actually progressing as a family, I think was, I think, really helpful for [young person]. And I think she found that it wasn't just her problem, it became our problem, right? And it became a family solution.”—*Parent 2

*1b. Therapeutic relationship.* Many said that a strong relationship with the therapist was instrumental in facilitating change. Parents that did not find FT-AN to be as helpful still described the therapeutic relationship as important. This enabled the young person and family to open up and utilise the space. Trust, specifically between the young person and therapist, was a key element in a strong therapeutic relationship, from the parents’ perspective. As young people developed faith in the therapist, they began to trust in the information and advice being given to them.

*“There was a sort of feeling of a big feeling of trust. I think she felt like she was in safe, she just felt like she was in safe hands. And so, and that sort of developing relationship of trusting someone, without sort of becoming too dependent, you know”—*Parent 4

*“As we progressed into the, going to see [therapist] more, I felt like  [young person] started to trust what she was saying more, and believing that what she said was true. So, then he was eating more, he stopped the exercising as that was continually told to him, that the exercising was no good”—*Parent 7

A second key aspect of the therapeutic relationship described was the therapist’s ability to bring their personality into the sessions while maintaining clear professional boundaries. Participants felt that an important part of the therapeutic relationship was balancing the challenging content and focus on eating with lightness and humour, which they perceived as being influenced by the therapist’s personality.

*“It wasn't some big drama, and [therapist] would make it very, very calm and cool and quite funny*.“*—*Parent 4

“[*Therapist] was -- without her personality and the way that she handled it, the way she prepared, the connection she got with [young person]. Fabulous. Absolutely fabulous… It was her that made it what it was. Made it a success.”—*Parent 10

*1c. Peer support.* Participants described connection with peers in a similar situation to be influential for both parents and for young people. Having a shared experience with others was felt to reinforce that they were not alone in the journey. Parents felt that connecting with others provided a place of understanding that could not have been achieved in other relationships.

“*It was positive [coming to clinic] I suppose in a way, because he knew that he wasn't the only one, he wasn't on his own*”*—*Parent 7

“I *think having a sister who had been through the same thing and was offering up advice and support, and whilst I'd rather they didn't both have been affected, it was helpful to have someone else that really got it*.”*—*Parent 19

Similarly, peer support was thought to be missing from participants who didn’t find treatment as helpful. There was a sense that having peer connection may have provided a key connection to facilitate change.

“*And I did wonder about - because I mentioned in the last session, group therapy, if there was a way for her to talk to other people, you know, who are in her situation, because she doesn't want to talk to, you know, us.”*—Parent 9

2. Strong foundations

The second overarching theme ‘*strong foundations*’ reflected the idea that pre-existing strong family bonds and proactive efforts prior to the commencement of treatment created a favourable starting point for change. This was then promoted by a therapeutic context in which young people and families felt emotionally held and contained. This theme encapsulates how families used pre-existing strengths and knowledge giving people a ‘*head start’*, the support of ‘*knowledgeable professionals’* in helping recognise the reality of having an eating disorder, creating a ‘c*ontaining’* environment in therapy through predictability, consistency and boundaries, whilst also allowing for ‘f*lexibility’* by meeting the need of each young person and family.

*2a. Head start—pre-existing family strengths.* Several parents spoke of a pre-existing strong family bond as a factor enabling change. There was a sense that having positive relationships entering treatment allowed young people to access more of the information and be more open to change.

*“But the fact we had a good relationship with her meant she cooperated rather than not. Yeah, I think because, a relationship with her was good. And we come, we as family, you know, we're not perfect, but we communicated. It just helps the process. Yeah, we were supportive”—*Parent 20

Parents also reported conducting their own research and implementing strategies at home while waiting to access treatment.

*“I think we did a lot of work ourselves as a family to read and stuff like that because ... and reached out to friends who went through similar situation”—Parent 2*

*2b. Knowledgeable professionals.* This sub-theme captured participants’ experiences of safety and trust in professionals, which were fostered through receiving treatment with a specialist service offering a structured treatment programme with clearly defined expectations. For some participants being accepted into the service provided evidence that things were not okay.

“*The fact she was going through the programme actually, for her, reinforced that something was not right*”*—*Parent 15

Being accepted to the service provided families with the knowledge that there are people to help them and guide the family to a better place. For some participants this took some of the weight of responsibility off them, freeing up mental capacity to be open to change and new learning on how to navigate the situation they were in.

“*I just really loved it because I felt like I'm not doing this by myself. I've got this other very responsible, expert, professional person who's in, for this period, is in our family too. And it's kind of saying you're doing a good job well done, you know, carry on, what we're going to do to make things better. And that was really, really helpful.”—*Parent 14

“*There was total trust. Okay, sort of you're in charge now. You tell us. And then we could come here, and no one gets told off. No one, you know, it's not stressful, but let's all just do what we can and listen to each other and then we'll be okay. It's just trusting, trusting in that I think was key.*”*—*Parent 11

Alongside feeling safe in the knowledge that a specialist team has oversight of their young person and family, being under the service provided an authority figure outside of the family reinforcing that there was a serious medical problem that needed addressing.

*“I think because [the therapist] was medically advised, it was coming from [the therapist] than me. Because it's, it's different when the parents trying to tell a child what could be happening when they actually hear it from somebody that I suppose has the full knowledge. It makes them realise that what I was saying is actually true.”—*Parent 12

*2c. Containment through balancing consistency with flexibility—taking a person-centred approach.* There was a sense that the predictability, consistency and expectations of the treatment provided to young people and their family in the early stages of treatment worked to create change. However, this needed to be balanced with some flexibility and holding the bigger picture in mind. Parents spoke of young people being responsive to the boundaries and structure of FT-AN, particularly the regularity of eating, early in treatment.

*“She's quite responsive to being told what to do … we had the [meal] plan. That plan was really useful, because we could then say, this is what you've got to do. And actually, she responded fairly well to that … there was a lot of, you've got to have your snack, you've got every … your meal is this, because you wouldn't always remember, or she would be deliberately skipping*”*—*Parent 15

“*It felt very regular and mostly on time, you know, obviously there were moments when things had to be changed and stuff, but there was a sort of beautiful consistency to it”—*Parent 4

Alongside the boundaries and clear expectations of FT-AN, participants described experiencing the boundaries of the therapeutic space itself as containing and fostering change.

“*I think having sort of a dedicated time and space to talk about some really difficult stuff, but knowing someone was listening, knowing someone was massively experienced, you know, and had evidence to help you through that, and just the kindness we were afforded, really”—*Parent 19

“*[young person] was in a safe space to open up, and then it gave [young person] the encouragement to obviously voice what was going on with [them]. Whereas I think when it's kind of one on one with a parent, [young person] doesn't want to open up. But I think in that environment it gave [young person] the confidence to do it.”—*Parent 12

While structure and guidance were generally considered helpful, participants spoke of needing to balance this with flexibility. Parents felt that although the clear expectations and firmness provided structure and a path for recovery, at the same time there was a need to listen and be responsive to the young person and family needs. Parents felt that holding the bigger picture in mind meant some flexibility was required and ensured the approach was kind and created a space for the adolescent voice. It was said that this helped the young person not feel that treatment was too relentless, and they were being treated as a whole person, not just the illness.

*“I could see, now actually, the way to get her better is to let her have a go at doing this herself… that, you know, letting go a bit, I think was really, really important, and fortunately, [young person] responded to that.”—*Parent 19

“*There were probably some times where she didn't eat as much as we wanted her to, but we kind of took that as a yeah, okay, we'll give you that one. But, you know, as long as just keeping the overall picture in our minds as opposed to being battling out this one specifically, so yeah.”—*Parent 15

3. Commitment

In this broad theme, parents spoke of the importance of the perceived commitment from all family members to get better, which was largely linked to internal or relational factors outside of therapy. This theme captures the idea from parents that, from their perspective, there was often a *‘wake-up call’* for young people in recognising the seriousness of the illness, and that parents perceiving the young person *‘getting on board’* with treatment was crucial for change.

*3a. Wake-up Call*. Parents perceived that young people often experienced a moment of clarity into the seriousness of the situation. This was felt to be a key foundation for change. Participants said that they noticed young people typically experiencing this early in treatment, often at the point of being accepted into the service and being diagnosed with an eating disorder.

“*The initial assessment with [therapist], the doctor, was very significant for [young person] because even though I had said literally everything that he said to her because I knew from a very medical family myself, and I'd obviously kind of read about anorexia and I knew about it, so I told her the consequences. Didn't penetrate at all. When he said it to her, she was shocked, and she started to take it really seriously. It's kind of like, really flipped a switch in her head*.”*—*Parent 14

“*For us, it was just getting in through the door, really… I think the turning point was before we arrived. So, I think it was just us realising there was a problem and getting her to agree to come*”*—*Parent 6

For others, they perceived this moment of clarity arising from early therapeutic conversations about the physical health risks of eating disorders and, specifically, being underweight.

*“[Therapist] made it very clear to the both of us right at the beginning how, you know, your, your body eats muscle and it will [eat] muscle from your heart, it'll eat muscle from your brain, and then the not making, decision making, you know, the judgement part shrinks. And that was a real shock because, you know, we were just sort of desperately worried about her, but [young person] really took that on board… She was frightened by that. And, so I think, yeah, I think we were lucky in the sense that she sort of got on board to do the eating plan”—*Parent 4

*“I think, for all of us, the reality of the health side of it and the importance of what they were trying to make us do took a little while for all of us to kind of really understand what that meant. But I think once we did, I think that helped us to move forward to the treatment.”—*Parent 2

*3b. Getting on board.* Participants perceived that young person’s motivation and openness to treatment was a key factor for change. For some participants they perceived their young person to be on board from the very beginning of therapy.

“*My daughter, luckily, completely accepted right from the off that the health professionals were right in what they were saying, and that she needed to make the changes*”*—*Parent 3

“*She almost decided immediately that the meal plan she was given that was almost her control, that she was going to follow that meal plan to the letter.”—*Parent 16

Some parents perceived an internal drive within the young person towards succeeding and doing well. This was described as serving recovery well in therapy.

*“She got ill very quickly because she was so determined to do it properly. But she got better very quickly because she was equally determined to recover quickly”—*Parent 13

“*If she's given a list of things, she will do it… Generally, her thing is she sets herself a challenge and she will do it. And that's why I knew that she would eventually eat at school because she had said that she would.”—*Parent 14

Other parents spoke about how they perceived their child as not particularly motivated at the beginning of treatment and that this needed to develop over time with their support.

“*I don't think [young person] will have done the changes if by herself. She wouldn't have the energy at the beginning and I think then she didn't just want it. So, I think it was really important that it was family and involved. And we were all planning the recovery.”*—Parent 22

4. Both/and rather than either/or

This broad theme highlights the necessity of maintaining a delicate balance to facilitate change. Parents described the need to carefully navigating different aspects of treatment to ensure the right equilibrium. This included *‘taking risks’*, finding *‘balance between the physical and emotional*, and *‘keeping normality going’* to allow families to take more responsibility for problem solving together without the support of treatment sessions.

*4a Taking risks.* Several parents spoke of the importance of positive risk taking and dignity of risk for recovery. There was a sense that taking the leap when a situation arose, enabled the young person (and parent) to push themselves and realise their strength. While taking risks did not always pay off, there was always important learning from these events. A parallel process was also described whereby young people taking risk also enabled parents to take risks towards recovery.

*“[young person] went on a school trip to Spain for a week, and [young person] was very wobbly about eating then. But when [young person] got back, [young person] was significantly better at home.”—*Parent 14

Although positive risk taking for some was felt to be a crucial turning point, this needed to be done cautiously and in collaboration with the young person. It was felt that, while safe risk taking was crucial for some, not all would be able to cope. There was an importance in understanding the individual and when to take risks.

*“We've learned that when people are in recovery from anorexia, going on holiday is an absolute no-no, it was horrific. [Therapist] did try and gently warn us that it may not be the best idea. And in fact, we haven't had a family holiday since, because we've realised it's too much pressure… we've learned that, actually, we can't force that*.”*—*Parent 8

Part of positive risk taking included spacing out treatment session. Participants generally described the usefulness of spacing out sessions across treatment and that the therapists created a good timeline for this. As sessions moved further apart this was generally seen as a helpful way to support change. Parents described it as enable the family to take more responsibility for change and manage positive risk taking themselves and for this to be modelled by the therapist.

*“I think at the beginning it was absolutely right. I think in the middle, when we were allowed a bit of space and when we were beginning to sort of trust [young person] to sort of do things. And that was about right. So, it's good that it was more intensive beginning it was okay that it spread out a bit.”—*Parent 11

“*We obviously for a good few months, was every week. And then you could see that she didn't want for attend every week and plus also, from our point of view, not a lot can change sometimes in a week. So, if we had a little bit longer [between sessions], then we could kind of see more where she was at. So that was all taken onboard. And that's how we did it. We then kind of went to every two weeks, and then we actually left it, I think, every three to four weeks, so it just kind of gave us that little bit longer to work on these things.”—*Parent 12

*4b Balance between the physical and emotional. *Participants highlighted the need for a holistic approach to facilitate change. They described that successful change occurred when the therapist was able to strike a balance between addressing the young person’s overt eating disorder symptoms and exploring the underlying emotional world. Parents perceived that finding the right balance was fluid rather than static, with the therapeutic focus shifting in response to the young person’s needs in the moment. Several participants described the balance shifting on a session-by-session basis rather than following at a predetermined transition point in therapy from the symptom management to the emotional exploration.

“*I think he had a good balance because some weeks it would be more focused on emotions and other weeks it would be focused on actions. It kind of -- he [therapist] kind of read her right as into what to deal with.”—*Parent 12

“*She, it was a safe place for her to kind of discuss things. And, you know, the talks would sort of take different turns, depending on what came up and the rest of it, so I mean, it was very, emotional support was, it was a huge part of it*”*—*Parent 4

Not all participants felt the right balance had been struck between physical and emotional support. Where this balance was not found, participants typically voiced that they imagined a better balance would have been helpful. There was a sense from parents that if more focus had been given to the young person’s emotional world, they would have made a more holistic recovery, rather than just a physical one. These parents were not always sure what exactly needed to be addressed; however, there was a sense that something was missing from the treatment.

“*What I understood, is we need to put [young person] in a better place, so then we can start with a psychological* *treatment... we put her in a healthy weight, but just when the psychological treatment should have a start, is when the treatment finished*.”—Parent 22

*“I think maybe she could have benefited from some one-on-one sessions. To kinda look further -- I don't know to have a look, at least into what's happening, why it's happening. Because we never really did that, we just tried to address the behaviour rather than why it's happening*.”*—*Parent 9

*4c Keeping normality going.* Participants said that keeping in touch with “reality” of everyday life was imperative for change to occur. They spoke of the importance of young person being able to engage in a life outside of the eating disorder during the therapeutic journey. Normality provided an escape and distraction for young people and ensure they continued to build their life (and identity) separate to the eating disorder.

*“She was really good at keeping some normality and keeping some things to look forward to, because him and her daughter are best friends from, like, we had our kids together, and we took turns to look after them together, and so she would just plan for him to go over there for weekends, and he would really look forward to it. And it would just give him a sort of break from it all. And a lot of the behaviours would kind of rein in a bit.”—Parent 1*

*“Her brother treated her no different from the start… I think that was a benefit, and I don't think that was ... I don't think that was something they discussed. I think that was probably my son's way of dealing with it, that actually she's still my sister.”—Parent 16*

5. Strengthening family connection

This theme reflected participants’ experiences of therapy as a process that enhanced their understanding of young person’s difficulties and equipped them with skills to respond in more supportive and validating ways. Parents described gaining confidence in taking an active role in their child’s recovery, while doing it in a way that the young person could experience as supportive. Improved communication and strengthened family connection were perceived as allowing families to navigate difficult situations more effectively and facilitate change.

*5a Parental understanding, skills and confidence.* Parents spoke of gaining new ways of understanding their young person and validating the difficulties they faced, which allowed them to better support them in their treatment journey. Contrastingly, those that described FT-AN as unhelpful, shared that they didn’t experience any greater understanding.

*“I probably started to listen to her more, which is interesting. Allowed me to see her as an adult.”—Parent 6*

*“I think once we learned a little bit more, we could understand why. And then the focus was more on encouraging her to eat, to be -- you know, to validate her feelings. That was a huge step for us. We didn't understand validation at all. Yeah, it was just completely alien so that -- it did help so much. It was a real turning point for us.”*—Parent 8

“*We were helped -- I mean, the validation was a really key point, we didn't really understand what that meant at the first, and obviously we got our heads around it.*”—Parent 10

“*I think finding better ways of talking to each other was part of it. And also, just knowing each other better. So, knowing when someone is in a difficult situation, as opposed to just cross about something. So, learning the differences around that and being able to describe that and talk about it, so that was quite helpful*.”—Parent 11

“*[I had to] change my way of thinking rather than the eating disorder consuming everything. It was looking at it a different way. So yeah, it definitely opened my eyes to a lot of things as well”*—Parent 12

Participants described FT-AN as helping them gain a feeling of confidence within the family. This enabled them to support and guide their young person and take an active role in helping them change in a way that the young person could experience as supportive.

*“I think, for us, the turning point was her actually us standing more up to [the illness behaviours] and [young person] understanding that she had to do it… And us trying to get through that stage of, you know, you just there's no discussion, you need to get on and do this”*—Parent 2

*“I think it gives sort of parents more traction to do the difficult things that need to be done, and the child can see it as being, you know, they're not being parents aren't being nasty or naggy or aggressive or any of these things, they're doing what they need to do to help them get better. So, by being altogether in that initial bit to sort of set out how that works and what the different roles was crucial, I would say.”*—Parent 19

Parents described the importance of gaining knowledge and insight into navigating eating disorders through therapy.

“*There were specific slides and information, bullet points, that were informative. And certainly, when you were the early stages of what the heck's going on with our child, that was, that was helpful.”*—Parent 10

*“We needed to be empowered to know what to do. And that's what we got from you, really. I didn't know I could tell her what to eat, for example, I didn't. I just didn't know. So, we were so cautious and so freaked out that it was like we just needed you to say, make her do this and she'll get, you know. I thought, okay, I can do that.”*—Parent 6

*5b Improved communication.* Participants that experienced treatment as helpful typically shared that, through FT-AN, they felt family relationships and communication had improved, allowing families to better navigate difficult situations. This, in turn, helped to create change.

*“I think it did sort of solidify my relationship with [young person]. I think that having, having, going through something like that, and especially we're a big family and you've got four kids, and they all need you, and they all want to have that time. Having that time, just with [young person] was very, was special, and I think because she was getting so much of it from the session, as was I, we sort of, it was bonding over a shared experience.“*—*Parent 4*

Participants that did not find FT-AN helpful, shared improved communication, although did not experience this as influential for change.

*“We were kind of forced to talk about things, because often she says I don't want to talk to you about this, etcetera, but in therapy have no choice but to talk. So, it was helpful in that aspect.”*—*Parent 9*

## Discussion

This qualitative study provides insights into parent and caregiver perceptions of factors that influence change during Family Therapy for Anorexia Nervosa (FT-AN). Five overarching themes were generated during the analysis—*Not alone, Strong foundations, Commitment, Both/and not either/or, and Strengthening family connections*—highlighting both facilitators of positive change and areas for potential improvement in treatment delivery.

Findings were generally consistent with existing literature, particularly with research examining young people’s experiences of change in FT-AN [35, 37]. Across participants, the importance of family involvement was emphasised. Even parents who reported dissatisfaction with FT-AN recognised the value of family inclusion, particularly given young people live within the context of their family. The therapeutic alliance emerged as critical, with parents emphasizing the importance of a knowledgeable, engaging, and trustworthy therapist who fosters a space for collaborative working. These findings align with concepts such as epistemic trust [27], relational containment [28], and broader evidence linking therapeutic relationships to improved outcomes [29, 31, 54].

Flexibility and dignity of risk were also identified as essential, especially in later treatment phases. Parents appreciated a gradual shift from structure to adaptability, which supported ongoing engagement. The need for increased positive risk taking from young people and parents was described as helping in building confidence within individuals and the family system, and the importance of making structural shifts to the treatment (e.g. spacing out sessions) helped this. This fits with the theoretical underpinnings of FT-AN, which promotes an expert yet warm therapeutic stance [3, 4, 12, 53]. It also highlights the delicate balance between structure and responsiveness required to maintain therapeutic momentum and the importance of shifting the therapeutic relationship across treatment. The use of formulation has been emphasised in revisions of FT-AN and FBT [4, 11, 12] and may provide a practical framework from which clinicians and families can collaborate on finding the balance between firmness and flexibility.

Of note, parental experiences of FT-AN were not universally positive. Two parents expressed concerns regarding an overemphasis on physical health at the expense of emotional needs, and a need for a greater understanding of the illness. These perspectives suggest some families may need more psychoeducation and a more integrated approach to balancing the behavioural and psychological targets. This fits with one criticism in the literature that family interventions can be perceived as too parent-focused and rigid, particularly in the early phases [21, 26].

Nevertheless, most parents described FT-AN as empowering—facilitating increased confidence, improved communication, and better boundary-setting. This echoes findings on parental self-efficacy, which has been shown to be associated with improved outcomes [9]. This finding emphasises the importance of ensuring clinicians take a stance of “*treatment with*”, not “*treatment of*”, the family. When the balance between firmness and flexibility was achieved, parents said their child’s recovery was supported both physically and emotionally. Several parents highlighted the impact of the therapist’s personal style, such as the use of appropriate humour and warmth, which helped shift treatment from a solely problem-focused frame to a more holistic and human experience.

An additional common thread between parent and young person accounts was the value of maintaining life beyond the eating disorder. Peers played a dual role by offering both normalization and a break from the intensity of treatment. Another shared theme was the occurrence of a moment of clarity or "awakening", often early in treatment, where young people came to accept the diagnosis and were able to engage more fully in the recovery process. These moments were seen by parents as crucial turning points.

A key difference between parent and young person perspectives was the emphasis placed on parental empowerment. Parents emphasised their need to gain confidence, better understand the illness, and acquire practical skills to manage their young person’s illness behaviours—elements they felt directly influenced change.

Finally, the findings echo research on broader family-based interventions for adolescent anorexia nervosa, which emphasises the importance of a strong therapeutic relationship, predictability, flexibility, and positive risk-taking in intensive family treatments [55–59], multi-family therapy [16–18, 60], and, to a degree, treatments for adolescent bulimia nervosa [61]. This suggests potential transdiagnostic mechanisms across family-informed models, including therapist qualities, parental empowerment, collaborative alliance, and flexibility.

Together, these findings support the continued development of nuanced, collaborative, and responsive family treatments for eating disorders anchored in strong therapeutic alliances and tailored to the diverse needs of both young people and their families.

### Strengths and limitations

This study has several strengths. Firstly, including the parent voice is particularly important given that they are vital within FT-AN. Studies have typically reported combined parent and young person experiences, making this study particularly useful in understanding the unique perspective of parents and caregivers. Secondly, participants were not excluded based on treatment outcome. This is useful as not every family has positive outcomes from FT-AN. These voices are equally important to capture alongside families with positive outcomes to identify similarities and differences in experience, as well as highlighting areas of improvement for treatment models.

Despite the study offering useful insights, there are several limitations to consider. Although the sample size is appropriate for qualitative research, it captured a relatively homogenous group from a small geographical area that received treatment at the same centre. Most participants were white, cisgendered women, with no participant identifying as neurodivergent. The voices of male caregivers were underrepresented (n = 5/23, 21.7%) and would be useful to explore further alongside the experience of families in other services and across more diverse groups. Furthermore, member checking was not included in the study meaning the analysis may be overly biased by researcher views. Additionally, less than half of the eligible families took part in the study, which might be indicative of selection bias. While there were no significant differences in young person age, treatment length or weight changes between participating and non-participating families, it is possible that there were differences not reported, such as treatment experience or completion. While all families who were discharged from treatment during the recruitment period were approached, regardless of outcome, it is likely that those who had a better outcome were more likely to engage in research with the team, meaning the data may be skewed towards those who had a positive experience of FT-AN. It will be important for future research to address these limitations, particularly by hearing from individuals who had a poorer response to FT-AN and those from more diverse backgrounds and treatment settings.

Lastly, most of the research team work in the recruiting service and have been part of developing the FT-AN model. This will have inevitably influenced the data analysis process. It would be useful to generate data from different clinics with researchers of differing experiences to shed new light on these findings.

## Conclusion

This study contributes to the growing body of qualitative research highlighting the complex, multifaceted nature of change in FT-AN. The findings emphasise the value of strong therapeutic relationships, family involvement, and a flexible approach that involves positive risk taking, tailored to the evolving needs of the family. While many parents described feeling more confident and better equipped to support their young person, the experiences of those who found FT-AN less helpful point to areas where treatment can be strengthened—particularly around balancing emotional wellbeing alongside physical recovery, and ensuring families are supported in understanding the illness from the outset. These insights build on existing theory and practice, and highlight the importance of a collaborative, relational, and adaptable therapeutic approach when working with families navigating adolescent anorexia nervosa. The current study highlights the need for further development and evaluation of more targeted, emotion-focused strategies to be incorporated into FT-AN, particularly in the later phases of treatment.

## Supplementary Information

Supplementary material 1

## Data Availability

Data will be made available upon reasonable request.
