# Supplementary material for: What factors do parents/caregivers think impact change in family therapy for anorexia nervosa?: a qualitative study
Source: J Eat Disord. 2025 Nov 6;13:250. doi: 10.1186/s40337-025-01368-x (PMC12590658; doi:10.1186/s40337-025-01368-x)
Supplement: Supplementary file 1 — Supplementary material 1 [file 40337_2025_1368_MOESM1_ESM.pdf]

Supplementary Material

Table S1 | The six phases of Reflexive Thematic Analysis and the associated involvement of each member of the research team.

Analysis Phase	Process	Analyser Involvement
1. Data familiarisation	Data were read repeatedly, understanding is rich and complex, and initial impressions are noted.	PJ engaged in data familiarisation and shared initial impressions with JB and AK.
2. Coding	Data were systematically coded- focusing on semantic meaning and remained close to participants reports.	PJ and AK coded the data separately, identifying topics within the data.<br><br>PJ and AK reviewed codes and discussed them, including identification of initial impressions of themes.
3. Initial theme generation	Initial themes were generated from codes and coded data, with similar and related topics being clustered.	PJ and AK generated initial themes.
4. Theme review and refinement	Initial themes were then reviewed independently in relation to topics and dataset, themes are edited and reviewed until they fit the data.	PJ reviewed the coding and initial themes and then generated themes that appeared to best fit the data.
5. Defining and naming themes	Each theme is refined and developed through naming and writing of the theme definition.	PJ named themes and wrote descriptions.
6. Producing the report	Report writing provided further opportunities for refinement.	PJ selected the illustrative quotes, and all authors reviewed and agreed. PJ then produced the manuscript, which was reviewed by JB.

Quality Assurance & Researcher Reflexivity

Interviews were conducted by two members of the research team, JB and AK. JB is a clinical psychologist with extensive clinical, theoretical and research experience in eating disorders and FT-AN. AK is a research assistant with no clinical experience of FT-AN but extensive theoretical knowledge. The trainee, PJ, joined the research team following data collection. PJ has some clinical and research experience of eating disorders and FT-AN. No research member disclosed personal experience of eating disorders or FT-AN. All members have an interest in eating disorders. JB and AK presently work in an eating disorder service. JB holds a PhD exploring multi-family therapy for eating disorders which AK was also a member of the research team for. PJ has worked in eating disorder services previously, including a placement during the course of the research, and completed previous research in the field of eating disorders. The research team were aware that their experiences would influence their interpretation of the data. For example, there is potential for PJ to attend more to aspects of the data that were consistent with their experiences from their own FT-AN clinical work.

A supervision session was held with JB prior to beginning analysis. This allowed the trainee to discuss this position entering the analysis phase. A reflective research diary (appendix E) was maintained throughout the trainee's involvement, to remain open to potential bias and assumptions being made throughout analysis.

Coding was completed independently. When both coders had analysed several interviews, a meeting was held to discuss codes and cross-check for differences in codes and to reflect on positionality. Coding was then completed, and initial themes were generated and revised over two hour-long meetings.
